# Supplementary material for: Patients’ and carers’ priorities for cancer research in Aotearoa/New Zealand
Source: PLoS One. 2023 Aug 22;18(8):e0290321. doi: 10.1371/journal.pone.0290321 (PMC10443847; doi:10.1371/journal.pone.0290321)
Supplement: Attachment — Submitted filename: Patients and carers priorities for CA research.pdf [file pone.0290321.s002.pdf]

PLOS ONE

Patients' and carers' priorities for cancer research in Aotearoa/New Zealand

--Manuscript Draft--

**Manuscript Number:**	PONE-D-23-10085
**Article Type:**	Research Article
**Full Title:**	Patients' and carers' priorities for cancer research in Aotearoa/New Zealand
**Short Title:**	Cancer research priorities
**Corresponding Author:**	Gabi U. Dachs, PhD<br>University of Otago Christchurch<br>Christchurch, Canterbury NEW ZEALAND
**Keywords:**	Cancer; survey; ethnicity; research priority; Māori
**Abstract:**	Background: Discrepancies have been reported between what is being researched, and what patients/families deem important to be investigated. Our aim was to understand research priorities for those who live with cancer in Aotearoa/New Zealand, with emphasis on Māori.<br>Methods: Adult outpatients with cancer and their whānau/family completed a survey (demographics, selecting keywords, free-text comments) at Christchurch hospital. Quantitative and qualitative data were evaluated using standard statistical and thematic analyses, respectively.<br>Results: We recruited 205 participants, including both tūroro/patients (n=129) and their whānau/family/carer (n=76). Partnership with Māori health workers enabled greater recruitment of Māori participants (19%), compared to the proportion of Māori in Canterbury (9%). Cancer research was seen as a priority by 96% of participants. Priorities were similar between Māori and non-Māori participants, with the keywords 'Cancer screening', 'Quality of Life' and 'Development of new drugs' chosen most often. Free-text analysis identified three themes; 'Genetics and Prevention', 'Early Detection and Treatment', and 'Service Delivery', with some differences by ethnicity.<br>Conclusions: Cancer research is a high priority for those living with cancer. In addition, participants want researchers to listen to their immediate and practical needs. These findings may inform future cancer research in Aotearoa.
**Order of Authors:**	Millie de Vries, BBiomedsc(Hons)
	Tiria Stewart
	Theona Ireton
	Karen Keelan
	Jennifer Jordan
	Bridget Robinson
	Gabi U. Dachs, PhD
**Additional Information:**	
**Question**	**Response**
**Financial Disclosure**<br><br>Enter a financial disclosure statement that describes the sources of funding for the work included in this submission. Review the submission guidelines for detailed requirements. View published research articles from *PLOS ONE* for specific examples.	Part-funding was obtained from the Cancer Society NZ (MV) and the Mackenzie Charitable Foundation (BAR, GUD). The remaining authors received no specific funding for this work.<br>The funders had no role in study design, data collection and analysis, decision to publish, or preparation of the manuscript.

Powered by Editorial Manager® and ProduXion Manager® from Aries Systems Corporation

This statement is required for submission and **will appear in the published article** if the submission is accepted. Please make sure it is accurate.<br><br>**Unfunded studies**<br>Enter: *The author(s) received no specific funding for this work.*<br><br>**Funded studies**<br>Enter a statement with the following details:<br>• Initials of the authors who received each award<br>• Grant numbers awarded to each author<br>• The full name of each funder<br>• URL of each funder website<br>• Did the sponsors or funders play any role in the study design, data collection and analysis, decision to publish, or preparation of the manuscript?<br>• **NO** - Include this sentence at the end of your statement: *The funders had no role in study design, data collection and analysis, decision to publish, or preparation of the manuscript.*<br>• **YES** - Specify the role(s) played.<br><br>* typeset	
**Competing Interests**<br><br>Use the instructions below to enter a competing interest statement for this submission. On behalf of all authors, disclose any competing interests that could be perceived to bias this work—acknowledging all financial support and any other relevant financial or non-financial competing interests.<br><br>This statement is required for submission and **will appear in the published article** if the submission is accepted. Please make sure it is accurate and that any funding sources listed in your Funding Information later in the submission form are also declared in your Financial Disclosure statement.<br><br>View published research articles from *PLOS ONE* for specific examples.	The authors have declared that no competing interests exist.

Powered by Editorial Manager® and ProduXion Manager® from Aries Systems Corporation

**NO authors have competing interests**<br><br>Enter: *The authors have declared that no competing interests exist.*<br><br>**Authors with competing interests**<br><br>Enter competing interest details beginning with this statement:<br><br>*I have read the journal's policy and the authors of this manuscript have the following competing interests: [insert competing interests here]*<br><br>* typeset	
**Ethics Statement**<br><br>Enter an ethics statement for this submission. This statement is required if the study involved:<br><br>• Human participants<br>• Human specimens or tissue<br>• Vertebrate animals or cephalopods<br>• Vertebrate embryos or tissues<br>• Field research<br><br>Write "N/A" if the submission does not require an ethics statement.<br><br>General guidance is provided below. Consult the submission guidelines for detailed instructions. **Make sure that all information entered here is included in the Methods section of the manuscript.**	This study was approved by the University of Otago Human Ethics Committee (H20/150) and all participants gave their consent in writing. The study was performed in accordance with the Declaration of Helsinki.

Powered by Editorial Manager® and ProduXion Manager® from Aries Systems Corporation

**Format for specific study types**<br><br>**Human Subject Research (involving human participants and/or tissue)**<br>• Give the name of the institutional review board or ethics committee that approved the study<br>• Include the approval number and/or a statement indicating approval of this research<br>• Indicate the form of consent obtained (written/oral) or the reason that consent was not obtained (e.g. the data were analyzed anonymously)<br><br>**Animal Research (involving vertebrate animals, embryos or tissues)**<br>• Provide the name of the Institutional Animal Care and Use Committee (IACUC) or other relevant ethics board that reviewed the study protocol, and indicate whether they approved this research or granted a formal waiver of ethical approval<br>• Include an approval number if one was obtained<br>• If the study involved *non-human primates*, add *additional details* about animal welfare and steps taken to ameliorate suffering<br>• If anesthesia, euthanasia, or any kind of animal sacrifice is part of the study, include briefly which substances and/or methods were applied<br><br>**Field Research**<br><br>Include the following details if this study involves the collection of plant, animal, or other materials from a natural setting:<br>• Field permit number<br>• Name of the institution or relevant body that granted permission	
**Data Availability**<br><br>Authors are required to make all data underlying the findings described fully available, without restriction, and from the time of publication. PLOS allows rare exceptions to address legal and ethical concerns. See the PLOS Data Policy and FAQ for detailed information.	Yes - all data are fully available without restriction

A Data Availability Statement describing where the data can be found is required at submission. Your answers to this question constitute the Data Availability Statement and **will be published in the article**, if accepted.<br><br>**Important:** Stating 'data available on request from the author' is not sufficient. If your data are only available upon request, select 'No' for the first question and explain your exceptional situation in the text box.<br><br>Do the authors confirm that all data underlying the findings described in their manuscript are fully available without restriction?	
**Describe where the data may be found in full sentences. If you are copying our sample text, replace any instances of XXX with the appropriate details.**<br><br>• If the data are **held or will be held in a public repository**, include URLs, accession numbers or DOIs. If this information will only be available after acceptance, indicate this by ticking the box below. For example: *All XXX files are available from the XXX database (accession number(s) XXX, XXX.).*<br>• If the data are all contained **within the manuscript and/or Supporting Information files**, enter the following: *All relevant data are within the manuscript and its Supporting Information files.*<br>• If neither of these applies but you are able to provide **details of access elsewhere**, with or without limitations, please do so. For example:<br><br>*Data cannot be shared publicly because of [XXX]. Data are available from the XXX Institutional Data Access / Ethics Committee (contact via XXX) for researchers who meet the criteria for access to confidential data.*<br><br>*The data underlying the results presented in the study are available from (include the name of the third party*	All relevant data are within the manuscript and its Supporting Information files.

and contact information or URL).<br>• This text is appropriate if the data are owned by a third party and authors do not have permission to share the data.<br><br>* typeset	
Additional data availability information:	

Manuscript

Click here to access/download;Manuscript;deVries 2023 Survey manuscript PLOS One v2.docx

Patients' and carers' priorities for cancer research in Aotearoa/New Zealand

Millie de Vries[1]*, Tiria Stewart[2]*, Theona Ireton[3], Karen Keelan[4], Jennifer Jordan[5,6], Bridget A Robinson[1,7], Gabi U Dachs[1].

[1]Mackenzie Cancer Research Group, University of Otago, Christchurch, New Zealand (NZ)

[2]Te Pūtahi Mātai Toto o Te Waipounamu, Christchurch Hospital, Christchurch, NZ (Nga Puhi, Ngāti Porou)

[3]Māori Health Services, Christchurch Hospital, Te Whatu Ora, Waitaha/Canterbury, Christchurch, NZ (Ngā Wairiki, Ngāti Porou)

[4]Te Aho o Te Kahu – Cancer Control Agency, Ministry of Health, NZ (Ngāti Porou)

[5]Psychological Medicine, University of Otago, Christchurch, NZ

[6]Specialist Mental Health Service Clinical Research Unit, Te Whatu Ora, Waitaha/Canterbury, NZ

[7]Canterbury Regional Cancer and Haematology Service, Te Whatu Ora, Waitaha/Canterbury, Christchurch, NZ

* These authors contributed equally to this work.

To be submitted to *PLOS One*

Abstract:

Background: Discrepancies have been reported between what is being researched, and what patients/families deem important to be investigated. Our aim was to understand research priorities for those who live with cancer in Aotearoa/New Zealand, with emphasis on Māori.

Methods: Adult outpatients with cancer and their whānau/family completed a survey (demographics, selecting keywords, free-text comments) at Christchurch hospital. Quantitative and qualitative data were evaluated using standard statistical and thematic analyses, respectively.

Results: We recruited 205 participants, including both tūroro/patients (n=129) and their whānau/family/carer (n=76). Partnership with Māori health workers enabled greater recruitment of Māori participants (19%), compared to the proportion of Māori in Canterbury (9%). Cancer research was seen as a priority by 96% of participants. Priorities were similar between Māori and non-Māori participants, with the keywords 'Cancer screening', 'Quality of Life' and 'Development of new drugs' chosen most often. Free-text analysis identified three themes; 'Genetics and Prevention', 'Early Detection and Treatment', and 'Service Delivery', with some differences by ethnicity.

Conclusions: Cancer research is a high priority for those living with cancer. In addition, participants want researchers to listen to their immediate and practical needs. These findings may inform future cancer research in Aotearoa.

Māori terms and translation:

Aotearoa (New Zealand); Waitaha (Canterbury); Ōtautahi (Christchurch); Te Whatu Ora (weaving of wellness, Health New Zealand); mate pukupuku (cancer); he aha ō whakaaro (what are your thoughts); whānau ((extended) family, based on whakapapa, here also carer); tūroro (patients) (alternative terms used: whānau affected by cancer or tangata whaiora (person seeking health)); wahine (female); tāne (male); rongoā (traditional healing); te reo (Māori language); whakapapa (genealogy); mokopuna (descendent); tikanga (methods, customary practices); hui (gathering)

Background

Cancer is a significant health burden in Aotearoa/New Zealand. Every year over 25,000 New Zealanders find out that they have cancer, and over 9,000 die from their disease [1]. Patterns of cancer registrations have changed over the years, with some cancers increasing (e.g. liver, pancreatic) and other cancers decreasing (e.g. stomach, lung) [1,2]. Mortality has reduced significantly for many cancer types over the last thirty years (data 1988 vs 2018: 165.2 vs 114.0 deaths per 100,000 [1]), due to reduction of risky behaviours (eg smoking), intensive screening and improved treatments, but counterbalanced by an aging population and increased rates of some comorbidities (eg obesity and diabetes) [3]. However, not all patients benefit, and ethnic disparities are evident along the entire cancer continuum [2]. Māori are about 20% more likely to develop cancer, and twice as likely to die from cancer than non-Māori [2].

The New Zealand Cancer Action Plan (Te Mahere mō te Mate Pukupuku o Aotearoa) 2019-2029 was developed to address these disparities and to ensure that all New Zealanders experience equitable cancer outcomes [4]. Improvements in cancer survival are incremental, and successful strategies are evidence-based and research-led. Cancer research in Aotearoa is largely led by clinical and scientific researchers. Due to Aotearoa's small population size, clinical research also often partners with international commercial clinical trials, but there are issues with these internationally-driven clinical paradigms which are underpinned by assumptions such as that all genders and ethnicities will respond similarly to treatments, or have similar beliefs about treatment and research [5]. Yet we know little about the research priorities of those who live with cancer, the tūroro/patients and their whānau/families. Under-representation of indigenous populations [6], such as Māori, in research is common and problematic, as their views on research priorities are unknown.

Research priorities of have been investigated in international studies [7, 8, 9], using a variety of approaches and prioritisation methods. Formal methodologies have been developed to achieve this, but none appear to be clearly advantageous for priority setting in Aotearoa [10]. One recent approach, the James Lind Alliance (JLA) method has been used for setting research priorities for the National Health Service in the United Kingdom [11]. The JLA approach uses a combination of surveys and workshops that involve patients, carers and health professionals to nominate the top unsolved research questions.

Using the JLA method, an analysis of the priorities of patients, clinicians and research communities has identified important mismatches [8]. The patient-clinician partnership prioritised education, training and service delivery, whereas both commercial and non-commercial trials prioritised drug development and other treatments [8].

Our study set out to determine the cancer research priorities of people living with cancer in Ōtautahi/Christchurch, Aotearoa. As limited data are available regarding cancer research priorities in Aotearoa [12, 13], we chose to adopt a simple survey format to gauge feedback from our community. Using structured and semi-structured questions, we aimed to determine the broad priorities of people living with cancer, how these may differ by ethnicity, sex and whether survey respondents were tūroro/patients or their whānau/family.

Methods

Ethics and ethnicity: This study was approved by the University of Otago Human Ethics Committee (H20/150). Ethnicity was self-declared, referring to cultural identity, and can contain individuals with more than one ethnic affiliation. Ethnicity data was collected using the 2013 census questionnaire. We used a prioritised ethnic classification system: individuals are classified as Māori if Māori was self-reported as one of the ethnic groups in any ethnicity field. Everyone else was grouped under the term non-Māori as a comparator. All collected data was deidentified and individuals are not identifiable.

Recruitment: Participants included tūroro/patients with cancer and their whānau/family, friends or carers. Participants were recruited in-person by a medical student (MT) or Māori health worker (TS) in the Oncology Unit or the South Island Bone Marrow Transplant Unit of Christchurch Hospital, respectively (Ōtautahi/Christchurch, Aotearoa/New Zealand), between November 2020 and July 2021. Christchurch hospital is the main tertiary hospital on the South Island of Aotearoa/New Zealand. Participants were provided with an information sheet, and consented to completing a simple paper survey. Inclusion criteria were: aged ≥ 18 years, tūroro/patients with a confirmed diagnosis of cancer, and/or member/s of their whānau/family or friend/carer/support person; tūroro/patients and whānau/family consented independently. The only exclusion was the inability to provide informed consent.

Survey and data collection: The simple 2-page survey “Mate Pukupuku: He aha ō whakaaro?” (“Cancer: What are your thoughts?”) was created specifically for this study in consultation with Māori (KK), and following discussions with clinicians at Christchurch Hospital (BR) and scientists at the University of Otago Christchurch (GD). A copy of the survey is available upon request from the corresponding author.

The survey comprised four sections, with section 1 collecting information on demographics (age, sex, ethnicity) and cancer type (for tūroro/patients only). In section 2 participants indicated priority rating of four broad research areas that broadly span the cancer continuum from cancer prevention, to diagnosis and prognosis of cancer, to reducing side effects of cancer treatment, and finally improving survival outcomes. Priorities were rated on a 4-point scale from ”very low” to “very high”. In section 3, participants were provided with a list of 44

cancer-related keywords, compiled by the scientists and clinicians in the author list. Of these keywords, any number could be chosen as being important, with participants circling words to indicate their choice of research priorities. The final section was a free-text question "What else do you think is important for cancer researchers to study?", inviting any additional input.

Hard copy paper surveys were completed by participants usually during their clinical visit at Christchurch hospital. Participants could either fill the survey in themselves or had help from the medical student or Māori health worker. Participant survey data were anonymised and each participant was allocated a unique research code. De-identified answers were transcribed onto an Excel spreadsheet, and stored as password-protected electronic files on a secure server.

Data analysis: This study followed a mixed methods approach. Quantitative data was analysed using descriptive statistics for categorical variables with GraphPad Prism 9. For qualitative data (free-text question), inductive thematic analysis and coding methods were used [14]. Codes identified from multiple readings of the raw data were grouped into categories independently by two researchers (MT, GD). Any differences were discussed and agreement reached. Categories were then synthesised into themes and sub-themes.

Results

Study cohort:

We recruited 205 participants, including both tūroro/patients (n=129) and their whānau/family/support (n=76) (**Table 1**). The average age was 59.7 years for tūroro and 53.1 years for whānau, and 60% were wahine/female. Our partnership with Māori health workers enabled greater recruitment of Māori participants (19%) compared to the proportion of Māori in the region (9.4% [15]). The majority of participants categorised as non-Māori identified as NZ European (69%), then Samoan (2.4%), with the remainder consisting of ethnicities with fewer than five participants each. As expected, the most common cancer types were breast, colorectal and prostate cancer [1].

Participants answered three types of questions for the survey: rating research priorities, choosing (most) important keyword from a list of cancer-related keywords, and adding further priorities as free text.

Rating research areas:

Participants rated four broad research areas from 'very low' to 'very high' priority (**Figure 1**). The vast majority (96%) of participants rated research of all four research areas (*Cancer Prevention, Diagnosis/Prognosis of Cancer, Reducing Side Effects,* and *Improving Outcomes from Cancer Treatment*) as 'high' or 'very high', with *Improving Cancer Outcomes* being marginally higher priority than others.

Cancer-related keywords:

The second question provided participants with a list of 44 cancer-related keywords from which any number could be chosen as being important. For all participants together, a median of 12 keywords was chosen by participants (range 0-44), and 12% chose all 44 keywords. 'Cancer screening' was the top priority (72% of participants), followed by 'bowel cancer' and 'breast cancer' (57% and 56% respectively) and 'development of new drugs' (56%). The keywords 'equity' (20%), 'smoking' (24%) and 'alcohol' (28%) were rarely chosen.

We were interested to see whether these priorities (by keywords) were different between Māori and non-Māori participants, with **Table 2** showing the top 10 chosen keywords by

ethnicity. When considering choices made by 60% or more of the participants, Māori participants chose 'quality of life', 'breast cancer' and 'Māori health', whereas non-Māori participants chose 'cancer screening', with the remaining keywords chosen by fewer than 60% of participants (**Table 2**).

We also investigated whether keyword choices differed between tūroro/patients with cancer and their whānau/family, or between wahine/females and tāne/males. Both tūroro and whānau chose 'cancer screening' as their top priority (74% and 68%, respectively). Aside from specific cancer types (bowel, breast and prostate cancer), it was interesting to see that patients more often chose 'new drug development' (57%) and 'cancer prevention' (56%), whereas non-patients more often chose 'quality of life' (59%) and 'reducing side effects' (55%). Similarly, both wahine and tāne chose cancer screening as top priority (72% and 73%, respectively), followed for wahine by breast cancer (63%), bowel cancer (60%) and side effects (59%), and for tāne followed by development of new drugs (52%), inherited cancers (52%) and side effects (51%).

Free text answers:

The third question of the survey invited participants to write down 'What else…', and was analysed qualitatively. From these free-text answers (n=106/205 participants), three main themes were identified: '*Why did I get cancer*?' (genetics and prevention), '*Can you detect cancer earlier and treat it better*?' (early detection and treatment), and '*Can you look after our patients better*?' (service delivery), each with several subthemes. Examples of de-identified participant comments are shown below.

Theme 1. Why did I get cancer?

Subtheme 1.1 Cancer risks and causes: Numerous participants expressed their anguish at not knowing why they, themselves, or their whānau, developed cancer. Many tried to find a reason why it happened, possibly in order to prevent others getting cancer or to blame something. Some stated that they lived a healthy life and did everything right, yet they developed cancer.

"I still have no idea of what may be the cause of my cancer. It was heart breaking because I made the decision to look after myself by eating well and exercise…" (CS137)

Putative environmental cancer risks were mentioned frequently, including *"nitrate in water"* (*CS019*), *"estrogen in rivers"* (*CS025*), *"hormone replacement therapy"* (*CS041*), *"cleaning products"* (*CS140*) and *"dental metals"* (*CS123*). Cancer incidence in Aotearoa as a whole or in specific locations and age groups, was deemed important to investigate (*"thirties aged people with bowel cancer ..in Westport"* (*CS003*), *"why Canterbury NZ has the highest rate of bowel cancer/capita in the world"* (*CS025*)).

Subtheme 1.2 Nutrition: Nutrition and diet were mentioned as potential risk factors, but also as putative preventative measures (*"Food (what I can/can not have), diet" (CS193)*, *"I am especially interested in learning more about diet" (CS019).* Participants provided very limited details for their answers.

Subtheme 1.3 Familial cancer and genetics: The inherited risk of developing cancer was seen as requiring further investigation, including specific genetic syndromes (*"Lynch syndrome"*). This was likely driven by a need to understand whether tūroro/patients inherited their cancer and the possible effect on their children and future generations. A mindset was apparent in older tūroro/patients, that genetic testing was helpful for the next generation, and less for themselves.

"To search up the link in family genes to see where it begins" (CS188)
"DNA (Family traits) inherited cancer for immigrants separated from family history" (CS128)

Subtheme 1.4 Prevention: Several participants cited the need for research into cancer prevention, although no details were provided (*"preventing it in the first place" (CS129)*). Not only prevention of the primary cancer, but also prevention of cancer recurrence was mentioned (*"alleviate recurrence"* (*CS176*)).

Subtheme 1.5 Rare cancers: Participants mentioned the need for research into rare cancers, often in relationship to their own diagnosis or that of their loved one. A need for more information on these unusual cancer types was expressed (*"Research into less common cancers - getting more awareness"* (*CS104*)).

Theme 2. Can you detect cancer earlier and treat it better?

Subtheme 2.1 Screening and early detection: This was an area of high importance to many participants. Some participants felt that their general practitioners missed the early signs of cancer and that the required testing was not done, leading to late diagnosis and poor prognosis. Hence, a *"more consistent screening programme"* and *"new methods for early detection"* (*CS116*) were listed. In addition, *"painless testing"* (*CS177*), was thought important.

"More. GP & A&E Testing before too late for diagnosis" (CS199)

"Leaving the ambulance at the bottom of the cliff will never be the best method; Screening early is the best method" (CS071)

As cancer incidence increases with age, most screening programs have a minimum age cut-off. However, some cancers are missed in those who are defined too young for screening, which was a concern for participants.

"I am sure the researchers are doing a fantastic job with all their researching but I do think that a lot of screening like bowel and breast cancer and many others, the age to test should be brought in for the younger generations as well" (CS036)

Subtheme 2.2 Complementary and holistic treatments: Over a quarter of Māori participants mentioned this subtheme, demonstrating their wish for further information and research in this area. Alternative and complementary treatments mentioned included *"natural medicines"* (CS177), *"herbal treatments"* and *"cannabis" (CS173)*, *"exercise" (CS104)* and *"music"* (CS187). Spirituality and holistic therapy were important themes.

"Complementary and alternative therapies, cannabis and vitamin C along with Māori Health and rongoā treatments" (CS020)

"Hospitals require to have facilities that offer holistic environments that ease, calm, relax, prepare patients physically, mentally, spiritually" (CS059)

The need for additional information on alternative or complementary therapies was clear, indeed clear information of effectiveness of all therapies was important, and trust in the treating physician was particularly vital.

"Trust & education on what is available for alternative medication for our tūroro" (CS197)

"For doctors and treatment to take into account faith and an holistic approach to treatment. To be able to be open minded and support a patient's right to take the journey however they see fit. To look at the use of alternative therapies and diet" (CS064)

Subtheme 2.3 New drugs and treatments: Many participants mentioned the need for better or *"painless treatment"* (*CS177*), as well as a need for treatments with fewer side effects.

"How to reduce side effects of chemotherapy/nausea" (CS016)

The need for more research into specific new drugs included *"immunotherapy" (CS004)* and *"Keytruda" (CS015)*. Participants expressed a need for *"more drug trials" (CS011)* and *"development of new cancer drugs" (CS119)*.

Subtheme 2.4 International standards and collaborations: Participants wanted world-class treatment and were concerned that some in Aotearoa might be missing out. For this reason, participants supported greater collaboration with other international researchers and clinicians, suggesting that this would bring expertise and access to the latest treatments to Aotearoa.

"Access to clinical trials from all NZ centres - international/Australian trials" (CS104)

"More research and communicate to other area, cities and countries who have more experience about it, more successful cases that they have done in past" (CS086)

Theme 3. Can you look after our patients better?

The health delivery system was an important theme, and whether it met the needs of the people it means to serve. This theme in particular showed the crossover between research wanted and support sought.

Subtheme 3.1 Role of health provider: Information provided during clinical visits is often overwhelming. Participants commented on the approach of their general practitioner or oncologist to delivering the diagnosis. A common concern was difficulty in understanding medical terminology and a lack of the right tools for this.

"The way we are told the results when the bad news is delivered" (CS178)

"Layman's terms if the person does not understand; fully understood; repeat if in doubt" (CS018)

Participants at times felt lost in the system, some describing it as chaotic, with a lack of continuity between services. Participants reported finding it hard to find support.

"The process once cancer is diagnosed - I believe the process is flawed and causes delays… it can feel like there are too many involved and that treatment of the patient becomes a bit like being on a production line" (CS136).

Subtheme 3.2 Access to care & finances & amenities: Finances were a key concern for participants (*"more national health assistance"* (*CS046*)), which is likely linked to time away from work or losing their job. Another major concern was the effect of cancer diagnosis and treatment on whānau/family, and how they were going to cope. The practical issues and daily stresses experienced by patients with cancer included (a perceived) lack of usable Wi-Fi (for Zoom communication with whānau) (*CS189*), public toilets (*CS179*) and parking during appointments (*CS204*).

"Transport sorted out for family to & from hospital (can be very costly for low income families) eg passes for family & support people" (CS180).

Subtheme 3.3 Public education and cancer information: Participants expressed the view that education and information should be more accessible, and that more resources for those diagnosed with cancer were needed in the community.

"A more comprehensive website or information so you don't have to search everywhere for information. A helpline that follows up once diagnosed, to give you support and talk through options, gives information." (CS166)

Subtheme 3.4 Emotional burden of cancer & survivorship & peer support: This subtheme was only mentioned by women, who felt that additional support and mentorship would help those living with cancer. It was felt that looking after tūroro at home was often challenging.

"Stress relief, for everyone concerned around family" (CS179)

"Emotional side of dealing with cancer and also what it does for families. Especially the spouses of those dealing with cancer. I feel they don't want to understand its harder for them to deal with" (CS167)

Subtheme 3.5 Culturally sensitive approaches: Some participants said they preferred to see 'someone like me', with regards to ethnicity, age, spirituality or religion, raising the requirement of clinical staff to be culturally aware and competent. This was not specific to Māori but included other ethnicities.

"Doctors/staff/nurses etc to be more informed/open to and with Māori health practices, ethnic background" (CS058)

"Information and health promotions focuses "pakeha" and it comes over as only pakeha get bowel cancer therefore many ethnic people don't know symptoms of bowel cancer such as bowel habit changes" (CS149)

Quantitative assessment of free-text entries:

To get further insight into participant's priorities, we also quantified the free-text entries. Overall, 52% of participants provided free-text answers, with 85% of Māori and 44% of non-Māori participants. Of the 33 Māori participants who provided free-text input, we identified 60 concepts (**Figure 2**). Māori participants commented most often on *Complementary and holistic treatments* (n=10, subtheme 2.2) and *Access to care & finances & amenities* (n=8, subtheme 3.2), followed by *Screening and Early detection* (n=6, subtheme 2.1) and *Culturally sensitive approaches* (n=6, subtheme 3.5). Of the 73 non-Māori participants who provided free-text input, we identified 124 concepts (**Figure 2**). Non-Māori participants wrote most often about *Cancer risks and causes* (n=21, subtheme 1.1) and *Screening and early detection* (n=21, subtheme 2.1), followed by *New drugs and treatments* (n=17, subtheme 2.3).

Of the 129 tūroro/patients, 48% completed free-text, compared to 59% of whānau/family. Tūroro most often commented on *Early detection* (n=17, subtheme 2.1), followed by *Complementary therapy* (n=14, subtheme 2.2) and *New drugs* (n=13, subtheme 2.3). Whānau commented equally often on *Cancer risks* (n=10, subtheme 1.1) and *Early detection* (n=10, subtheme 2.1).

Of the 123 wahine/females, 56% completed free-text, whereas 46% of tāne/males did. Wahine commented most often on *Early detection* (n=16, subtheme 2.1) followed by *Cancer risks* (n=15, subtheme 1.1), then *Complementary therapy* (n=14, subtheme 2.2). Tāne wrote about *Early detection* (n=11, subtheme 2.1) most often, followed equally by *Cancer risks* (n=7, subtheme 1.1), *Complementary therapy* (n=7, subtheme 2.1) and *New drugs* (n=7, subtheme 2.3).

Discussion

This study aimed to gain an understanding of research priorities from the perspective of those with a cancer diagnosis or their whānau/family in Aotearoa/New Zealand. Emphasis was placed on obtaining views from diverse participants, specifically from Māori, but also from tūroro/patients and whānau/family, and wahine/females and tāne/males. A close collaboration with Māori health workers enabled recruitment of over twice the proportion of Māori participants compared to the general population in Canterbury. The use of Te Reo Māori in parts of the survey and in reporting these results is done on purpose to enhance inclusivity of Aotearoa's indigenous population.

The survey used a mixed method approach, asking participants to rate the priorities of broad research areas, to select priorities from a list of cancer-related topics, and finally to expand on or provide free text comments on research priorities not already covered. Virtually all participants viewed cancer research as a very important mission, with largely similar priorities between Māori and non-Māori participants. Keywords 'Cancer screening', 'Quality of Life' and 'Development of new drugs' were chosen most often. Free-text analysis identified three themes; *'Genetics and Prevention'* (understanding environmental, genetic risks), *'Early Detection and Treatment'* (improving screening, treatments), and *'Service Delivery'* (information and support, culturally sensitive treatment).

Health disparities have been reported worldwide for indigenous and minority populations [16, 17]. In addition, it has been recognised that much of the research to date has lacked ethnic and other diversity, and there have been calls for a change in recruitment practices to ensure equitable participation [18]. It is vital to ensure appropriate reporting of health research involving indigenous people and not to, inadvertently, propagate bias [19]. For this reason, the CONSIDER statement has been developed, which is a framework for researchers that aims to 'strengthen research practices and reporting' and 'to support indigenous health equity' [18]. Our study has attempted to address these issues by consulting with Māori advisors from the start, by working in partnership with Māori, and by including te reo Māori in participant's narratives and tikanga Māori processes of engagement.

The majority of participants questioned why they had cancer, indicating that they supported more research on aetiology, and related to that, prevention. Participants were concerned

about the risks of (rare) heritable cancers. Attitudes to genetic testing, when mentioned, were positive, with examples of local celebrities (eg the musician Stan Walker) used to illustrate the utility of testing for whānau. Genetic testing is still seen as potentially contentious for Māori, due to genetic samples and whakapapa being taonga that don't just belong to the individual but have implications for whānau and iwi [20]. The absence of negative attitudes to genetic testing in this sample may be because they were facing life-threatening illness themselves with potential implications for wider whānau and mokopuna (descendent), hence the risk-benefit balance may be different for individuals in those circumstances.

Although we identified strong lived experience support for ongoing research into cancer screening and early diagnosis, it is recognised that screening, in particular, is complex and comes with its own controversies. These issues include false positive and false negative findings, potential harm from the test itself (e.g. radiation exposure), the possibility of overdiagnosis and how to handle incidental findings [21]. A current issue being debated in Aotearoa is the starting age of bowel screening, particularly as, in comparison to non-Māori, a greater percentage of bowel cancers in Māori occur before the age of 60 years, the current age of screening [22]. It is clear that further research is needed into availability of screening and improved early identification methods.

The areas of smoking and alcohol were seldom chosen as research priorities. This appears at odds with the high rates of smoking and alcohol (ab)use in Aotearoa [3]. The cancers most closely related to tobacco smoking and alcohol show high rates; in 2018 lung cancer had the highest cancer mortality rate (21/100,000) and liver cancer was also relatively high (3.4/100,000) [1, 2]. Some possible explanations for this apparent lack of interest may be that these behaviours are deemed to be already well understood as risk factors, or that stigma and blame surrounds these behaviours, or that it is too late for those who have the disease.

Quite apart from stigma towards cancers related to tobacco or alcohol-use amongst those affected by cancer, this stigma is also said to influence research in this area. Kamath [23] reports that this stigma hinders research funding applications, whereas some research areas with lower societal cancer burden, such as rare cancers, apparently tend to receive disproportionate attention. These issues represent an equity issue in Aotearoa.

It is of interest that only 20% of participants endorsed "equity" as a research priority; the lowest ranking of all priorities. It appears that this heading was not recognised by the participants. From free-text answers it seemed that equity was indeed of high priority but was placed under other guises. Indeed, many of the research areas mentioned (eg screening) and issues raised (eg service delivery) directly reflect equity matters. In future research, consideration should be given to elaborating on the meaning of the word to see whether that changes the current low ranking by those with lived experience, which is at odds with the priority placed on equity (in research) by researchers and research funders [24].

Participants were interested in incorporation of complementary or alternative treatments. There is increasing recognition of the benefits of incorporating holistic treatments alongside medical treatments for cancer [25, 26, 27]. Complementary medicine strategies are of interest in Aotearoa [28] and may improve quality of life of patients with cancer, but robust clinical data is sparse and the value for our population is unknown. The need for complementary/alternative therapies may also reflect a mistrust of modern medicine or the health services, or reflect a structural barrier in terms of how the health system has been designed [6].

Several participants felt strongly about spirituality and religion, which are well-known coping mechanisms for patients with cancer [29]. Spirituality recognises a sense of something greater than oneself, and can be viewed as separate to religion, or intimately linked. Specifically, Māori origins of spirituality are deeply embedded within their genealogy and ancestral heritage (whakapapa) [30], and should therefore be addressed as part of cancer support.

Consistent with this and the call for culturally sensitive treatment, there is increasing interest in researching the benefits of traditional Rongoā Māori practices to improve wellbeing for Māori patients [30]. It is important to note that Rongoā Māori is more than another complementary healing medicine, and is recognised as a holistic life practice, embedded within a Te Ao Māori (Māori world) cultural values framework [30]. Rongoā Māori is recognised by the NZ Māori Health Authority (Te Aka Whai Ora) and supported by the NZ Health Research Council [31].

Although the focus of this study was on cancer research priorities, a number of participants commented on areas of dissatisfaction with service delivery and current practises. The

comments (indirectly) addressed equity as the majority of the remarks reflected inequities related to ethnicity, socio-economic, education and health literacy. Our survey showed the value of an independent party talking directly with tūroro and whānau, such that they felt empowered to report problems and areas for improvement. Consistent with our findings, many previous studies have noted the complex issues involved in the initial communication and receipt of a cancer diagnosis, and the fact that understanding emerges over time, so the provision of information whenever the person is ready and needs it, is important [32, 33, 34]. Information on cancer produced specifically for different ethnicities or age groups could also be helpful.

The survey also providing the opportunity to comment on the need for practical support, such as financial aid, Wifi and parking. A cancer diagnosis negatively affects the financial wellbeing of most tūroro and their whānau due to treatment-related costs and loss of income, and varies by socioeconomic status [35]. These additional stresses make the cancer journey harder and impact on patients' survival [36, 37].

The strengths of this study include that, to our knowledge, it is the first study in Aotearoa identifying the views of tūroro and their whānau on priorities for cancer research. The partnership with Māori health workers contributed to the high participation of Māori in this study and the detailed comments provided.

There are a number of limitations of this study. First, although free text answers provided valuable additional insight, it is also clear that the (limited) words written down do not fully reflect the participants suggestions or emotions. Future research should include qualitative interviews which would allow more in-depth understanding of participants comments. A hui (gathering) process to gather Māori-rich information would be another vital avenue to consider. Another limitation is that the survey, being carried out at a major tertiary hospital, may not have captured priorities and aspirations for whānau living rurally.

In conclusion, cancer research was a high priority for those living with cancer, with support for research across the spectrum from aetiology and prevention, to better, more effective cancer treatments. It has become obvious, though, that many participants request that we, as researchers, listen to tūroro/patients and whānau/family regarding their immediate and practical needs. From their comments, there is clearly a need for further research into areas

of treatment satisfaction and unmet needs in service delivery, including the need for better information, culturally appropriate treatment and incorporation of complementary and Rongoā Māori practices to improve quality of life while living with cancer. These findings will inform future cancer research in Aotearoa.

Acknowledgements

Authors' contributions

MV and TS recruited and interviewed participants. TS, TI and KK provided Māori guidance. GD, KK and BR designed the study. JJ supported the thematic analysis and BR provided clinical guidance. GD coordinated the study and wrote the draft manuscript. All authors contributed to editing the manuscript and approved the submitted version.

Ethics approval and consent to participate

This study was approved by the University of Otago Human Ethics Committee (H20/150) and all participants gave their consent in writing. The study was performed in accordance with the Declaration of Helsinki.

Consent for publication

Not required.

Data availability

All data generated or analysed during this study are included in this manuscript.

Competing interests

The authors declare no conflict of interest.

Funding information

Funding was obtained from the Cancer Society NZ and the Mackenzie Charitable Foundation.

References

1. Historical cancer data. Cancer registration and death data for selected cancers from 1948 to the present (2022). https://www.health.govt.nz/publication/cancer-historical-summary-1948-2018; accessed 27 March 2023

2. He Pūrongo Mate Pukupuku o Aotearoa 2020, The State of Cancer in New Zealand 2020 (2021). https://teaho.govt.nz/static/reports/state-of-cancer-in-new-zealand-2020%20(revised%20March%202021).docx; accessed 27 March 2023

3. Annual update of key results 2021/22: New Zealand Health Survey (2022). https://www.health.govt.nz/publication/annual-update-key-results-2021-22-new-zealand-health-survey ; accessed 27 March 2023

4. New Zealand Cancer Action Plan 2019–2029 (2020). https://www.health.govt.nz/publication/new-zealand-cancer-action-plan-2019-2029; accessed 27 March 2023

5. National Academies of Sciences, Engineering, and Medicine; Policy and Global Affairs; Committee on Women in Science, Engineering, and Medicine; Committee on Improving the Representation of Women and Underrepresented Minorities in Clinical Trials and Research; Bibbins-Domingo K, Helman A, editors. Improving Representation in Clinical Trials and Research: Building Research Equity for Women and Underrepresented Groups. Washington (DC): National Academies Press (US); 2022, 2, Why Diverse Representation in Clinical Research Matters and the Current State of Representation within the Clinical Research Ecosystem. Available from: https://www.ncbi.nlm.nih.gov/books/NBK584396/

6. Carr, T., Arcand, L., Roberts, R., Sedgewick, J., Ali, A., Groot, G. The experiences of Indigenous people with cancer in Saskatchewan: a patient-oriented qualitative study using a sharing circle. *CMAJ Open* **8**(4), E852-E859 (2020).

7. Tsianakas, V., Maben, J., Wiseman, T., Robert, G., Richardson, A., Madden, P., Griffin, M., Davies, E.A. Using patients' experiences to identify priorities for quality improvement in breast cancer care: patient narratives, surveys or both? *BMC Health Serv. Res*. **12**, 271 (2012).

8. Crowe, S., Fenton, M., Hall, M., Cowan, K., Chalmers, I. Patients', clinicians' and the research communities' priorities for treatment research: there is an important mismatch. *Res. Involv. Engagem*. **1**, 2 (2015).

9. Hathaway, C.A., Siegel, E.M., Chung, C.H., Pabbathi, S., Vidrine, J., Vadaparampil, S., et al. Utilizing a large-scale biobanking registry to assess patient priorities and preferences for cancer research and education. *PLoS ONE* **16**(2), e0246686 (2021).

10. Background Consultation Documents (2018). https://www.hrc.govt.nz/news-and-events/background-consultation-documents/ accessed 27 March 2023

11. Nygaard, A., Halvorsrud, L., Linnerud, S., Grov, E.K., Bergland, A. The James Lind Alliance process approach: scoping review. *BMJ Open*. **9**(8), e027473 (2019).

12. O'Brien, I., Signal, L., Sarfati, D. Wide-ranging impacts reported by NZ cancer survivors: is supporting cancer survivor resilience a health sector role? *Support Care Cancer* **26**(4), 1207-1213 (2018).

13. Babashahi, S., Hansen, P., Sullivan, T. Creating a priority list of non-communicable diseases to support health research funding decision-making. *Health Policy* **125**(2), 221-228 (2021).

14. Braun, V., Clarke, V. Using thematic analysis in psychology. *Qual. Res. Psych*. **3**(2), 77-101 (2006).

15. 2018 Census place summaries. Canterbury Region (2021). https://www.stats.govt.nz/tools/2018-census-place-summaries/canterbury-region; accessed 27 March 2023

16. Dachs, G.U., Currie, M.J., McKenzie, F., Jeffreys, M., Cox, B., Foliaki, S., Le Marchand, L., Robinson, B.A. Cancer disparities in indigenous Polynesian populations: Māori, Native Hawaiians, and Pacific people. *Lancet Oncol*. **9**(5), 473-84 (2008).

17. Vaccarella, S., Lortet-Tieulent, J., Saracci, R., Fidler, M.M., Conway, D.I., Vilahur, N., Sarfati, D., Jemal, A., Mackenbach, J.P., Marmot, M.G., Straif, K., Wild, C.P. Reducing Social Inequalities in Cancer: Setting Priorities for Research. *CA Cancer J Clin*. **68**(5), 324-326 (2018).

18. Huria, T., Palmer, S.C., Pitama,. S, Beckert, L., Lacey, C., Ewen, S., Smith, L.T. Consolidated criteria for strengthening reporting of health research involving indigenous peoples: the CONSIDER statement. *BMC Med. Res. Methodol*. **19**(1), 173 (2019).

19. Sarfati, D., Garvey, G., Robson, B., Moore, S., Cunningham, R., Withrow, D., Griffiths, K., Caron, N.R., Bray, F. Measuring cancer in indigenous populations. *Ann. Epidemiol*. **28**(5), 335-342 (2018).

20. Hudson, M., Mead, A.T.P., Chagné, D., Roskruge, N., Morrison, S., Wilcox, P.L., Allan, A.C. Indigenous Perspectives and Gene Editing in Aotearoa New Zealand. *Front. Bioeng. Biotechnol*. **7**, 70 (2019).

21. Thomas, E.T., Del Mar, C., Glasziou, P., Wright, G., Barratt, A., Bell, K.J.L. Prevalence of incidental breast cancer and precursor lesions in autopsy studies: a systematic review and meta-analysis. *BMC Cancer* **17**, 808 (2017).

22. McLeod, M., Harris, R., Paine, S.J., Crengle, S., Cormack, D., Scott, N., Robson, B. Bowel cancer screening age range for Māori: what is all the fuss about? *N. Z. Med. J*. **134**(1535), 71-77 (2021).

23. Kamath, S.D., Kircher, S.M., Benson, A.B. Comparison of Cancer Burden and Nonprofit Organization Funding Reveals Disparities in Funding Across Cancer Types. *J. Natl. Compr. Canc. Netw*. **17**(7), 849-854 (2019).

24. Lacey, C., Lawrence, M., Paterson, C., Every-Palmer, S. Voices forgotten or a future of inclusion and equity: An Aotearoa New Zealand perspective on better publication of Indigenous mental health research. *Aust. N. Z. J. Psychiatry*. **56**(8):895-898 (2022).

25. Cadet, T., Davis, C., Elks, J., Wilson, P. A holistic model of care to support those living with and beyond cancer. *Healthcare* **4**(4), 88 (2016).

26. Lyman, G.H., Greenlee, H., Bohlke, K., Bao, T., DeMichele, A.M., Deng, G.E., et al. Integrative therapies during and after breast cancer treatment: ASCO endorsement of the SIO clinical practice guideline. *J. Clin. Oncol*. **36**(25), 2647–55 (2018).

27. Kristoffersen, A.E., Wider, B., Nilsen, J.V., Bjelland, M., Mora, D.C., Nordberg, J.H., Broderstad, A.R., Nakandi, K., Stub, T. Prevalence of late and long-term effects of cancer

(treatment) and use of complementary and alternative medicine in Norway. *BMC Complement. Med. Ther.* **22**(1), 1-21 (2022).

28. Vernon-Roberts, A., Denny, A., Day, A.S. Point Prevalence of Complementary or Alternative Medicine Use among Children Attending a Tertiary Care Hospital. *Children* (Basel). **10**(1), 132 (2023).

29. Selman, L.E., Brighton, L.J., Sinclair, S., Karvinen, I., Egan, R., Speck, P., Powell, R.A., Deskur-Smielecka, E., Glajchen, M., Adler, S., Puchalski, C., Hunter, J., Gikaara, N., Hope, J., InSpirit Collaborative. Patients' and caregivers' needs, experiences, preferences and research priorities in spiritual care: A focus group study across nine countries. *Palliat. Med*. **32**(1), 216-230 (2018).

30. Mark, G., Boulton, A., Kerridge, D. Rongoā māori is not a complementary and alternative medicine: rongoā māori is A way of life*. Int. J. Hum. Rights Ed*. **3**(1), 12 (2019).

31. Rongoā (2023). https://www.teakawhaiora.nz/our-work/rongoa/ accessed 27 March 2023

32. Melis, P., Galletta, M., Aviles Gonzalez, C.I., Contu, P., Jimenez Herrera, M.F. Experiencing communication related to knowing the cancer diagnosis and prognosis: A multi-perspective interpretative phenomenological study. *Eur. J. Oncol. Nurs*. **51**, 101904 (2021).

33. Anderson, J.N., Graff, J.C., Krukowski, R.A., Schwartzberg, L., Vidal, G.A., Waters, T.M., Paladino, A.J., Jones, T.N., Blue, R., Kocak, M., Graetz, I. "Nobody will tell you. You've got to ask!": An examination of patient-provider communication needs and preferences among black and white women with early-stage breast cancer. *Health Commun*. **36**(11), 1331-1342 (2021).

34. Golden, S.E., Ono, S.S., Thakurta, S.G., Wiener, R.S., Iaccarino, J.M., Melzer, A.C., Datta, S.K., Slatore, C.G. "I'm putting my trust in their hands": A qualitative study of patients' views on clinician initial communication about lung cancer screening. *Chest*. **158**(3), 1260-1267 (2020).

35. Kong, Y.C., Wong, L.P., Ng, C.W., Taib, N.A., Bhoo-Pathy, N.T., Yusof, M.M., *et al*. Understanding the financial needs following diagnosis of breast cancer in a setting with universal health coverage. *Oncologist* **25**(6), 497-504 (2020).

36. Sharples, K.J., Firth, M.J., Hinder, V.A., Hill, A.G., Jeffery, M., Sarfati. D., *et al*. The New Zealand PIPER Project: colorectal cancer survival according to rurality, ethnicity and socioeconomic deprivation-results from a retrospective cohort study. *N. Z. Med. J*. **131**(1476), 24-39 (2018).

37. Gurney, J., Stanley, J., McLeod, M., Koea, J., Jackson, C., Sarfati, D. Disparities in Cancer-Specific Survival Between Māori and Non-Māori New Zealanders, 2007-2016. *JCO Glob. Oncol*. **6**,766-774 (2020).

Figure legends

Figure 1. Research priorities of all participants by category.

Participants chose the priority for each of 4 broad categories in cancer research from 'very low' to 'very high'. The proportion of responses are shown for n=205 participants.

Figure 2. Thematic answers according to participants' ethnicity.

Participants' free-text entries were analysed by inductive thematic analysis and coding methods, with 3 broad themes and multiple subthemes identified. The number of responses under each subtheme are shown. Of 205 participants, n=106 participants provided comments, including 33 who identified as Māori and 73 who identified as non-Māori.

Tables

Table 1: Characteristics of tūroro/patients and their whānau/family cohort.

**Demographics**		**n=**	**%**
Cohort	*total*	*205*	*100.0*
Ethnicity	Māori	39	19.0
	Non- Māori	166	81.0
Age	<50 years	57	27.8
	50+ years	147	71.7
	not stated	1	0.5
Sex	Wahine/Female	123	60.0
	Tāne/Male	81	39.5
	Not stated	1	0.5
Participants	Tūroro/Patients	129	62.9
	Whānau/Family	76	37.1
Cancer types	*all*	*129*	*100.0*
	Breast	29	22.5
	Bowel	16	12.4
	Prostate	14	10.9
	Lung	9	7.0
	Lymphoma	8	6.2
	Melanoma	8	6.2
	Leukaemia	6	4.7
	other	37	28.7
	not stated	2	1.6

Table 2: Top chosen keywords according to ethnicity.

All participants	n=	%	Non-Māori participants	n=	%	Māori participants	n=	%
Total	*205*	*100.0*	*Total*	*166*	*100*	*Total*	*39*	*100*
Cancer Screening	148	72.2	Cancer Screening	125	75.3	Quality of Life	25	64.1
Bowel Cancer	117	57.1	Bowel Cancer	97	58.4	Breast cancer	24	61.5
Breast cancer	115	56.1	Side effects of treatment	93	56.0	Māori Health	24	61.5
Development of new drugs	114	55.6	Development of new drugs	92	55.4	Cancer Screening	23	59.0
Cancer prevention	111	54.1	Breast cancer	91	54.8	Inherited cancers	23	59.0
Quality of Life	111	54.1	Cancer prevention	90	54.2	Development of new drugs	22	56.4
Side effects of treatment	110	53.7	Quality of Life	86	51.8	Mental health and cancer	22	56.4
Prostate cancer	101	49.3	Prostate cancer	84	50.6	Cancer prevention	21	53.8
Cancer genetics	99	48.3	Cancer and immune system	80	48.2	Men's health	21	53.8
Pain relief and cancer	99	48.3	Diet and cancer	80	48.2	Bowel Cancer	20	51.3
			Pain relief and cancer	80	48.2	Cancer genetics	20	51.3
						Cannabis and cancer	20	51.3

Note: Participants could choose 0-44 keywords.

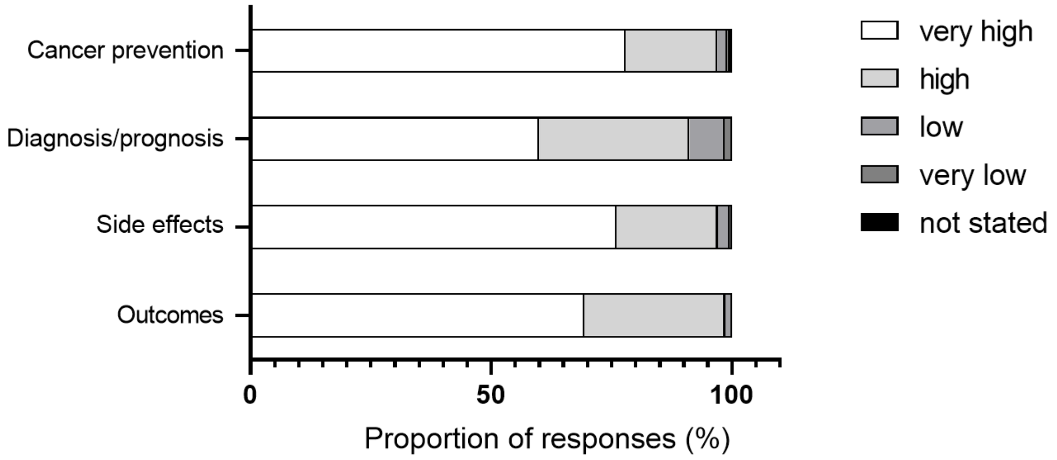
Cancer prevention
Diagnosis/prognosis
Side effects
Outcomes
0
50
100
Proportion of responses (%)
very high
high
low
very low
not stated

Figure 1

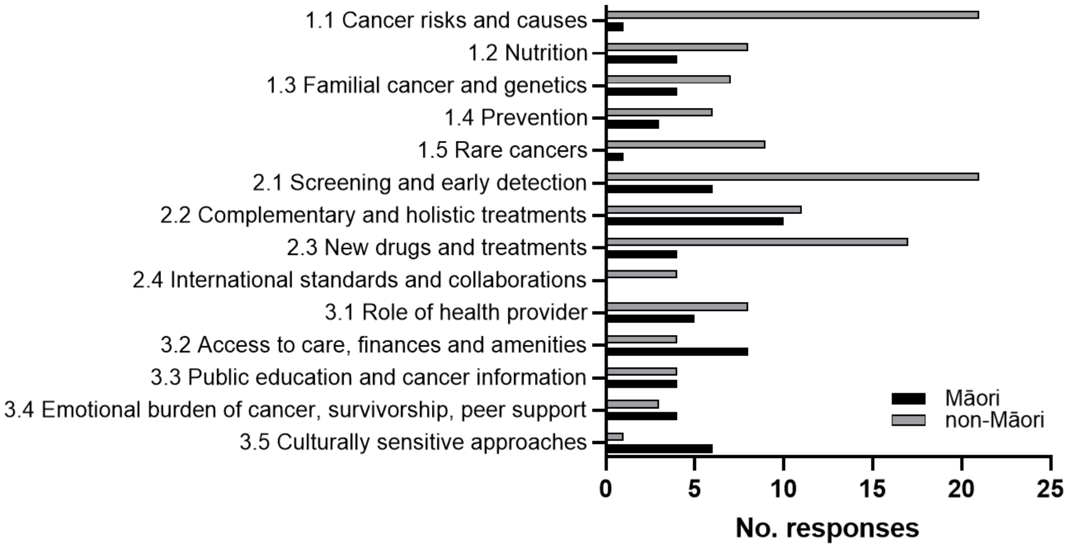
1.1 Cancer risks and causes
1.2 Nutrition
1.3 Familial cancer and genetics
1.4 Prevention
1.5 Rare cancers
2.1 Screening and early detection
2.2 Complementary and holistic treatments
2.3 New drugs and treatments
2.4 International standards and collaborations
3.1 Role of health provider
3.2 Access to care, finances and amenities
3.3 Public education and cancer information
3.4 Emotional burden of cancer, survivorship, peer support
3.5 Culturally sensitive approaches
Māori
non-Māori
0
5
10
15
20
25
No. responses

Figure 2
